# *N*-(3-Meth­oxy­benzo­yl)-4-methyl­benzene­sulfonamide

**DOI:** 10.1107/S1600536813019107

**Published:** 2013-07-17

**Authors:** S. Sreenivasa, B. S. Palakshamurthy, T. N. Lohith, N. R. Mohan, Vijith Kumar, P. A. Suchetan

**Affiliations:** a Department of Studies and Research in Chemistry, Tumkur University, Tumkur, Karnataka 572 103, India; b Department of Studies and Research in Physics, U.C.S., Tumkur University, Tumkur, Karnataka 572 103, India; c University College of Science, Tumkur University, Tumkur, India; d Soild State and Structural Chemistry Unit, Indian Institute of Science, Bangalore, India; e Department of Studies and Research in Chemistry, U.C.S., Tumkur University, Tumkur, Karnataka 572 103, India

## Abstract

In the title compound, C_15_H_15_NO_4_S, the dihedral angle between the benzene rings is 88.87 (1)°. In the crystal, adjacent mol­ecules form inversion dimers through pairs of strong N—H⋯O hydrogen bonds, generating *R*_2_^2^(8) loops. Two C—H⋯π inter­actions and an aromatic π–π inter­action [centroid–centroid separation = 3.8191 (1) Å] are also observed.

## Related literature

For a similar structure, see: Suchetan *et al.* (2010 ▶).
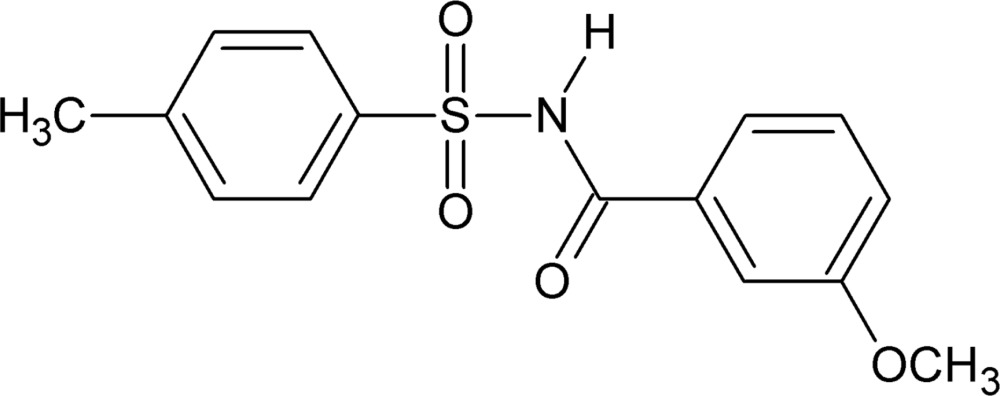


## Experimental

### 

#### Crystal data

C_15_H_15_NO_4_S
*M* *_r_* = 305.34
Triclinic, 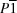

*a* = 9.2474 (7) Å
*b* = 9.6660 (6) Å
*c* = 9.8764 (8) Å
α = 70.268 (6)°
β = 64.052 (8)°
γ = 86.231 (5)°
*V* = 743.69 (11) Å^3^
*Z* = 2
Mo *K*α radiation
μ = 0.23 mm^−1^
*T* = 293 K
0.35 × 0.28 × 0.22 mm

#### Data collection

Bruker APEXII diffractometer
Absorption correction: multi-scan (*SADABS*; Bruker, 2009 ▶) *T*_min_ = 0.925, *T*_max_ = 0.950
11424 measured reflections
2610 independent reflections
2212 reflections with *I* > 2σ(*I*)
*R*_int_ = 0.037

#### Refinement

*R*[*F* ^2^ > 2σ(*F* ^2^)] = 0.039
*wR*(*F* ^2^) = 0.112
*S* = 1.06
2610 reflections
196 parameters
H atoms treated by a mixture of independent and constrained refinement
Δρ_max_ = 0.28 e Å^−3^
Δρ_min_ = −0.25 e Å^−3^

### 

Data collection: *APEX2* (Bruker, 2009 ▶); cell refinement: *APEX2* and *SAINT-Plus* (Bruker, 2009 ▶); data reduction: *SAINT-Plus* and *XPREP* (Bruker, 2009 ▶); program(s) used to solve structure: *SHELXS97* (Sheldrick, 2008 ▶); program(s) used to refine structure: *SHELXL97* (Sheldrick, 2008 ▶); molecular graphics: *Mercury* (Macrae *et al.*, 2008 ▶); software used to prepare material for publication: *SHELXL97*.

## Supplementary Material

Crystal structure: contains datablock(s) I, global. DOI: 10.1107/S1600536813019107/bg2511sup1.cif

Structure factors: contains datablock(s) I. DOI: 10.1107/S1600536813019107/bg2511Isup2.hkl

Supplementary material file. DOI: 10.1107/S1600536813019107/bg2511Isup3.cml

Additional supplementary materials:  crystallographic information; 3D view; checkCIF report

## Figures and Tables

**Table 1:** Hydrogen-bond geometry (Å, °) *Cg*1 and *Cg*2 are the centroids of the sulfonyl-bound and carbonyl-bound benzene rings respectively.

*D*—H⋯*A*	*D*—H	H⋯*A*	*D*⋯*A*	*D*—H⋯*A*
N1—H*N*1⋯O1^i^	0.79 (2)	2.14 (2)	2.920 (2)	170 (2)
C15—H15*B*⋯*Cg*1^ii^	0.96	2.77	3.576 (3)	141
C7—H7*A*⋯*Cg*2^iii^	0.96	2.94	3.753 (3)	143

Symmetry codes: (i) 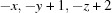; (ii) 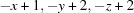; (iii) 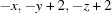.

**Fig. 1.**
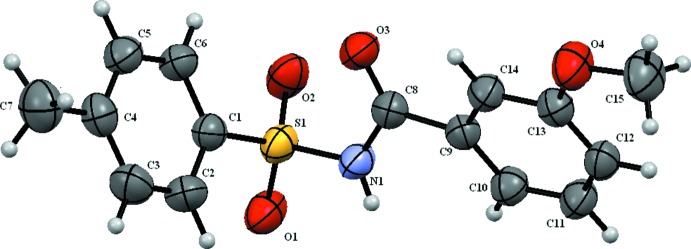
Molecular structure of the title compound, showing the atom-labeling scheme. Displacement ellipsoids are drawn at the 50% probability level.

**Fig. 2.**
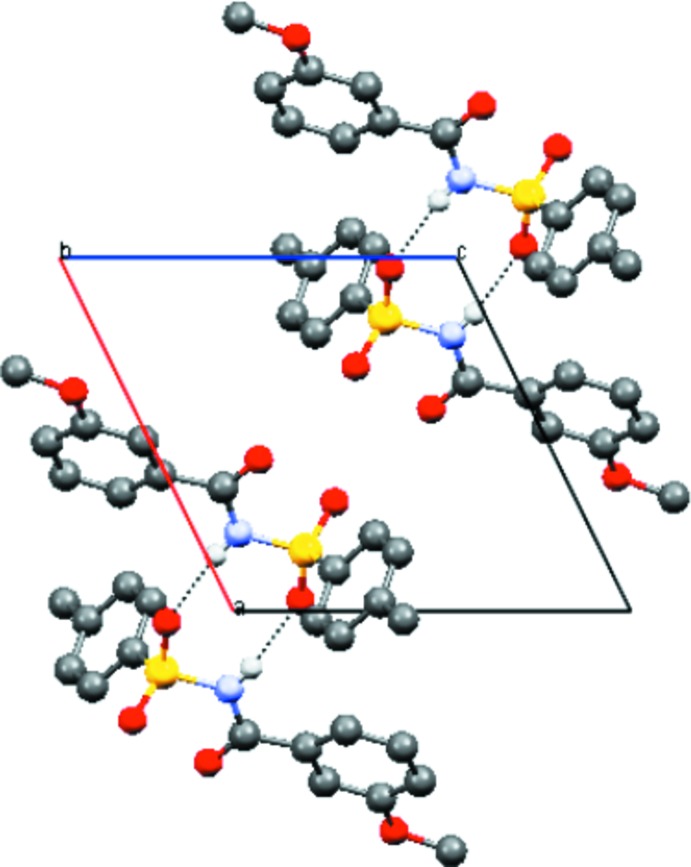
Molecular packing of (I) with hydrogen bonding shown as dashed lines. Carbon bounded hydrogen atoms are ommitted for clarity.

**Fig. 3.**
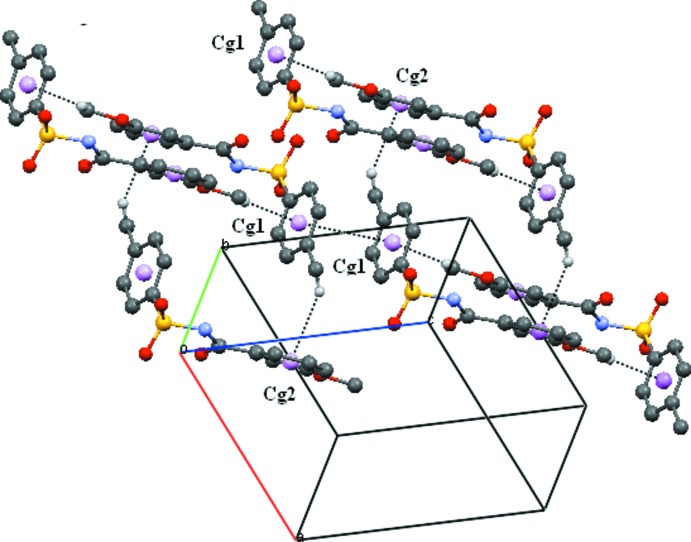
Display of C—H···π interactions and stacking of molecules through π-π interactions. Cg1 and Cg2 are the centroids of the sulfonyl bound and carbonyl bound benzene ring respectively. For clarity purpose, the hydrogen atoms not involved in hydrogen bonding are ommitted.

## supplementary crystallographic information

### Comment

As a part of our continued efforts to study the crystal structures of *N*-(aroyl)-arylsulfonamides (Suchetan *et al.*, 2010), we report here the crystal structure of the title compound (I) (Fig 1).

The conformation of the N—H bond in I is anti to the C=O bond in the side chain, similar to that observed in. *N*-(benzoyl)-4-methylbenzenesulfonamide (II, Suchetan *et al.*, 2010). Further, the conformation between the N—H bond and the *meta*-methoxy group in the benzoyl ring is anti.

The dihedral angle between the methyl-substituted benzene ring (maximum deviation from mean plane: 0.007 Å for C5) and the methoxy-substituted benzene ring (maximum deviation from mean plane: 0.005 Å for C13) is 88.87 (1)°. Adjacent molecules form inversion related dimers through strong N—H···O hydrogen bonds, generating *R*_2_^2^(8) loops (Fig 2). Two C—H···π interactions and an aromatic π-π interaction (centroid-centroid separation = 3.8191 (1) Å) are also observed in the structure (Fig 3).

### Experimental

The title compound was prepared by refluxing a mixture of 3-methoxybenzoic acid, 4-methylbenzenesulfonamide and phosphorous oxychloride (POCl_3_) for 2 h on a water bath. The resultant mixture was cooled and poured into ice cold water. The solid obtained was filtered and washed thoroughly with water and then dissolved in sodium bicarbonate solution. The compound was later reprecipitated by acidifying the filtered solution with dilute HCl. The compound obtained was filtered and later dried (Melting point: 405 K.) Colorless prisms of (I) were obtained from a slow evaporation of its ethanolic solution at room temperature.

### Refinement

The H atom of the NH group was located in a difference map and later refined freely. The other H atoms were positioned with idealized geometry using a riding model with C—H = 0.93 Å. All H atoms were refined with isotropic displacement parameters (set to 1.2 times of the *U*_eq_ of the parent atom).

### Crystal data

**Table:**

C_15_H_15_NO_4_S	*F*(000) = 320
*M**_r_* = 305.34	Prism
Triclinic, *P*1	*D*_x_ = 1.364 Mg m^−^^3^
Hall symbol: -P 1	Melting point: 405 K
*a* = 9.2474 (7) Å	Mo *K*α radiation, λ = 0.71073 Å
*b* = 9.6660 (6) Å	Cell parameters from 1123 reflections
*c* = 9.8764 (8) Å	θ = 2.4–25.0°
α = 70.268 (6)°	µ = 0.23 mm^−^^1^
β = 64.052 (8)°	*T* = 293 K
γ = 86.231 (5)°	Prism, colourless
*V* = 743.69 (11) Å^3^	0.35 × 0.28 × 0.22 mm
*Z* = 2	

### Data collection

**Table:**

Bruker APEXII diffractometer	2212 reflections with *I* > 2σ(*I*)
Radiation source: fine-focus sealed tube	*R*_int_ = 0.037
Graphite monochromator	θ_max_ = 25.0°, θ_min_ = 2.4°
phi and ω scans	*h* = −10→10
Absorption correction: multi-scan (*SADABS*; Bruker, 2009)	*k* = −11→11
*T*_min_ = 0.925, *T*_max_ = 0.950	*l* = −11→11
11424 measured reflections	3 standard reflections every 1 reflections
2610 independent reflections	intensity decay: 10%

### Refinement

**Table:**

Refinement on *F*^2^	Primary atom site location: structure-invariant direct methods
Least-squares matrix: full	Secondary atom site location: difference Fourier map
*R*[*F*^2^ > 2σ(*F*^2^)] = 0.039	Hydrogen site location: inferred from neighbouring sites
*wR*(*F*^2^) = 0.112	H atoms treated by a mixture of independent and constrained refinement
*S* = 1.06	*w* = 1/[σ^2^(*F*_o_^2^) + (0.0515*P*)^2^ + 0.194*P*] where *P* = (*F*_o_^2^ + 2*F*_c_^2^)/3
2610 reflections	(Δ/σ)_max_ = 0.046
196 parameters	Δρ_max_ = 0.28 e Å^−^^3^
0 restraints	Δρ_min_ = −0.25 e Å^−^^3^

### Special details

**Table:**

Geometry. All s.u.'s (except the s.u. in the dihedral angle between two l.s. planes) are estimated using the full covariance matrix. The cell s.u.'s are taken into account individually in the estimation of s.u.'s in distances, angles and torsion angles; correlations between s.u.'s in cell parameters are only used when they are defined by crystal symmetry. An approximate (isotropic) treatment of cell s.u.'s is used for estimating s.u.'s involving l.s. planes.
Refinement. Refinement of *F*^2^ against ALL reflections. The weighted *R*-factor *wR* and goodness of fit *S* are based on *F*^2^, conventional *R*-factors *R* are based on *F*, with *F* set to zero for negative *F*^2^. The threshold expression of *F*^2^ > 2σ(*F*^2^) is used only for calculating *R*-factors(gt) *etc*. and is not relevant to the choice of reflections for refinement. *R*-factors based on *F*^2^ are statistically about twice as large as those based on *F*, and *R*- factors based on ALL data will be even larger.

### Fractional atomic coordinates and isotropic or equivalent isotropic displacement parameters (Å^2^)

**Table:**

	*x*	*y*	*z*	*U*_iso_*/*U*_eq_	
HN1	0.159 (2)	0.588 (2)	0.975 (3)	0.051 (6)*	
C1	0.1161 (2)	0.8457 (2)	0.6955 (2)	0.0476 (5)	
C2	−0.0258 (2)	0.8795 (3)	0.8029 (2)	0.0613 (6)	
H2	−0.0909	0.8073	0.8997	0.074*	
C3	−0.0693 (3)	1.0213 (3)	0.7648 (3)	0.0662 (6)	
H3	−0.1646	1.0442	0.8371	0.079*	
C4	0.0256 (2)	1.1310 (2)	0.6209 (3)	0.0564 (5)	
C5	0.1653 (2)	1.0931 (2)	0.5154 (2)	0.0553 (5)	
H5	0.2292	1.1646	0.4174	0.066*	
C6	0.2129 (2)	0.9520 (2)	0.5511 (2)	0.0518 (5)	
H6	0.3084	0.9291	0.4791	0.062*	
C7	−0.0228 (3)	1.2854 (3)	0.5835 (3)	0.0798 (7)	
H7A	−0.1380	1.2822	0.6220	0.120*	
H7B	0.0286	1.3360	0.4696	0.120*	
H7C	0.0098	1.3368	0.6350	0.120*	
C8	0.3499 (2)	0.7247 (2)	0.8738 (2)	0.0482 (5)	
C9	0.3856 (2)	0.6830 (2)	1.0147 (2)	0.0446 (4)	
C10	0.3358 (2)	0.5448 (2)	1.1356 (2)	0.0513 (5)	
H10	0.2709	0.4755	1.1353	0.062*	
C11	0.3844 (2)	0.5127 (2)	1.2554 (2)	0.0554 (5)	
H11	0.3525	0.4203	1.3357	0.066*	
C12	0.4792 (2)	0.6145 (2)	1.2591 (2)	0.0519 (5)	
H12	0.5105	0.5910	1.3411	0.062*	
C13	0.5272 (2)	0.7517 (2)	1.1398 (2)	0.0508 (5)	
C14	0.4814 (2)	0.7857 (2)	1.0170 (2)	0.0512 (5)	
H14	0.5150	0.8777	0.9361	0.061*	
C15	0.6780 (3)	0.8309 (3)	1.2494 (3)	0.0782 (7)	
H15A	0.5879	0.8074	1.3536	0.117*	
H15B	0.7428	0.9162	1.2284	0.117*	
H15C	0.7421	0.7488	1.2462	0.117*	
N1	0.2204 (2)	0.6435 (2)	0.8917 (2)	0.0519 (4)	
O1	0.03105 (19)	0.56323 (17)	0.81816 (17)	0.0674 (4)	
O2	0.30852 (18)	0.65075 (17)	0.60995 (16)	0.0643 (4)	
O3	0.42784 (17)	0.82263 (17)	0.74840 (17)	0.0633 (4)	
O4	0.6206 (2)	0.86118 (18)	1.13085 (19)	0.0731 (5)	
S1	0.17181 (6)	0.66553 (6)	0.74433 (6)	0.05142 (19)	

### Atomic displacement parameters (Å^2^)

**Table:**

	*U*^11^	*U*^22^	*U*^33^	*U*^12^	*U*^13^	*U*^23^
C1	0.0453 (10)	0.0573 (12)	0.0412 (10)	0.0008 (8)	−0.0216 (8)	−0.0141 (9)
C2	0.0511 (12)	0.0712 (15)	0.0451 (11)	−0.0006 (10)	−0.0140 (9)	−0.0093 (10)
C3	0.0524 (12)	0.0811 (17)	0.0590 (13)	0.0133 (11)	−0.0192 (11)	−0.0256 (12)
C4	0.0553 (12)	0.0622 (13)	0.0619 (13)	0.0095 (10)	−0.0341 (11)	−0.0227 (11)
C5	0.0562 (12)	0.0537 (12)	0.0494 (11)	−0.0019 (9)	−0.0231 (10)	−0.0091 (9)
C6	0.0478 (11)	0.0584 (12)	0.0427 (10)	0.0014 (9)	−0.0168 (9)	−0.0134 (9)
C7	0.0820 (17)	0.0748 (17)	0.0900 (18)	0.0235 (14)	−0.0445 (15)	−0.0313 (14)
C8	0.0462 (10)	0.0508 (11)	0.0453 (11)	0.0046 (9)	−0.0213 (9)	−0.0124 (9)
C9	0.0416 (10)	0.0490 (11)	0.0425 (10)	0.0064 (8)	−0.0192 (8)	−0.0144 (8)
C10	0.0537 (11)	0.0480 (11)	0.0516 (11)	0.0014 (9)	−0.0252 (9)	−0.0133 (9)
C11	0.0625 (13)	0.0487 (12)	0.0494 (11)	0.0032 (9)	−0.0274 (10)	−0.0064 (9)
C12	0.0522 (11)	0.0606 (13)	0.0453 (11)	0.0097 (9)	−0.0257 (9)	−0.0163 (9)
C13	0.0486 (11)	0.0543 (12)	0.0526 (11)	0.0042 (9)	−0.0259 (9)	−0.0171 (9)
C14	0.0518 (11)	0.0492 (11)	0.0501 (11)	0.0008 (9)	−0.0254 (9)	−0.0094 (9)
C15	0.0851 (17)	0.0866 (18)	0.0832 (17)	−0.0041 (14)	−0.0573 (15)	−0.0237 (14)
N1	0.0531 (10)	0.0574 (11)	0.0395 (9)	−0.0067 (8)	−0.0228 (8)	−0.0048 (8)
O1	0.0801 (10)	0.0657 (10)	0.0571 (9)	−0.0210 (8)	−0.0398 (8)	−0.0038 (7)
O2	0.0778 (10)	0.0666 (10)	0.0472 (8)	0.0167 (8)	−0.0259 (7)	−0.0220 (7)
O3	0.0605 (9)	0.0685 (10)	0.0493 (8)	−0.0100 (7)	−0.0258 (7)	−0.0015 (7)
O4	0.0847 (11)	0.0708 (11)	0.0749 (10)	−0.0137 (8)	−0.0520 (9)	−0.0113 (8)
S1	0.0595 (3)	0.0537 (3)	0.0418 (3)	−0.0022 (2)	−0.0265 (2)	−0.0105 (2)

### Geometric parameters (Å, º)

**Table:**

C1—C6	1.381 (3)	C9—C10	1.394 (3)
C1—C2	1.383 (3)	C10—C11	1.378 (3)
C1—S1	1.750 (2)	C10—H10	0.9300
C2—C3	1.377 (3)	C11—C12	1.380 (3)
C2—H2	0.9300	C11—H11	0.9300
C3—C4	1.389 (3)	C12—C13	1.381 (3)
C3—H3	0.9300	C12—H12	0.9300
C4—C5	1.380 (3)	C13—O4	1.366 (2)
C4—C7	1.500 (3)	C13—C14	1.385 (3)
C5—C6	1.383 (3)	C14—H14	0.9300
C5—H5	0.9300	C15—O4	1.426 (3)
C6—H6	0.9300	C15—H15A	0.9600
C7—H7A	0.9600	C15—H15B	0.9600
C7—H7B	0.9600	C15—H15C	0.9600
C7—H7C	0.9600	N1—S1	1.6477 (16)
C8—O3	1.211 (2)	N1—HN1	0.79 (2)
C8—N1	1.388 (2)	O1—S1	1.4338 (15)
C8—C9	1.488 (3)	O2—S1	1.4199 (15)
C9—C14	1.386 (3)		
			
C6—C1—C2	120.7 (2)	C11—C10—H10	120.5
C6—C1—S1	120.07 (15)	C9—C10—H10	120.5
C2—C1—S1	119.19 (15)	C10—C11—C12	121.46 (19)
C3—C2—C1	119.2 (2)	C10—C11—H11	119.3
C3—C2—H2	120.4	C12—C11—H11	119.3
C1—C2—H2	120.4	C11—C12—C13	119.41 (18)
C2—C3—C4	121.5 (2)	C11—C12—H12	120.3
C2—C3—H3	119.3	C13—C12—H12	120.3
C4—C3—H3	119.3	O4—C13—C12	124.67 (18)
C5—C4—C3	117.8 (2)	O4—C13—C14	115.23 (18)
C5—C4—C7	121.7 (2)	C12—C13—C14	120.10 (18)
C3—C4—C7	120.5 (2)	C13—C14—C9	120.09 (18)
C4—C5—C6	121.92 (19)	C13—C14—H14	120.0
C4—C5—H5	119.0	C9—C14—H14	120.0
C6—C5—H5	119.0	O4—C15—H15A	109.5
C1—C6—C5	118.76 (19)	O4—C15—H15B	109.5
C1—C6—H6	120.6	H15A—C15—H15B	109.5
C5—C6—H6	120.6	O4—C15—H15C	109.5
C4—C7—H7A	109.5	H15A—C15—H15C	109.5
C4—C7—H7B	109.5	H15B—C15—H15C	109.5
H7A—C7—H7B	109.5	C8—N1—S1	123.04 (14)
C4—C7—H7C	109.5	C8—N1—HN1	122.9 (15)
H7A—C7—H7C	109.5	S1—N1—HN1	113.9 (15)
H7B—C7—H7C	109.5	C13—O4—C15	118.08 (17)
O3—C8—N1	120.27 (18)	O2—S1—O1	118.61 (10)
O3—C8—C9	123.41 (17)	O2—S1—N1	109.61 (9)
N1—C8—C9	116.31 (16)	O1—S1—N1	103.41 (9)
C14—C9—C10	120.02 (17)	O2—S1—C1	109.64 (9)
C14—C9—C8	116.76 (17)	O1—S1—C1	109.03 (9)
C10—C9—C8	123.10 (17)	N1—S1—C1	105.68 (9)
C11—C10—C9	118.92 (18)		
			
C6—C1—C2—C3	−0.4 (3)	C11—C12—C13—C14	−0.6 (3)
S1—C1—C2—C3	179.73 (17)	O4—C13—C14—C9	−179.59 (17)
C1—C2—C3—C4	0.2 (3)	C12—C13—C14—C9	0.8 (3)
C2—C3—C4—C5	0.7 (3)	C10—C9—C14—C13	−0.3 (3)
C2—C3—C4—C7	−178.8 (2)	C8—C9—C14—C13	−176.47 (17)
C3—C4—C5—C6	−1.4 (3)	O3—C8—N1—S1	4.2 (3)
C7—C4—C5—C6	178.2 (2)	C9—C8—N1—S1	−175.21 (13)
C2—C1—C6—C5	−0.2 (3)	C12—C13—O4—C15	2.4 (3)
S1—C1—C6—C5	179.63 (14)	C14—C13—O4—C15	−177.17 (19)
C4—C5—C6—C1	1.1 (3)	C8—N1—S1—O2	54.03 (19)
O3—C8—C9—C14	18.3 (3)	C8—N1—S1—O1	−178.56 (16)
N1—C8—C9—C14	−162.34 (17)	C8—N1—S1—C1	−64.03 (18)
O3—C8—C9—C10	−157.8 (2)	C6—C1—S1—O2	−7.39 (19)
N1—C8—C9—C10	21.6 (3)	C2—C1—S1—O2	172.45 (15)
C14—C9—C10—C11	−0.5 (3)	C6—C1—S1—O1	−138.75 (16)
C8—C9—C10—C11	175.44 (17)	C2—C1—S1—O1	41.08 (18)
C9—C10—C11—C12	0.7 (3)	C6—C1—S1—N1	110.66 (17)
C10—C11—C12—C13	−0.2 (3)	C2—C1—S1—N1	−69.50 (17)
C11—C12—C13—O4	179.91 (19)		

### Hydrogen-bond geometry (Å, º)

*Cg*1 and *Cg*2 are the centroids of the sulfonyl-bound and carbonyl-bound benzene rings respectively.

**Table:**

*D*—H···*A*	*D*—H	H···*A*	*D*···*A*	*D*—H···*A*
N1—H*N*1···O1^i^	0.79 (2)	2.14 (2)	2.920 (2)	170 (2)
C15—H15*B*···*Cg*1^ii^	0.96	2.77	3.576 (3)	141
C7—H7*A*···*Cg*2^iii^	0.96	2.94	3.753 (3)	143

Symmetry codes: (i) −*x*, −*y*+1, −*z*+2; (ii) −*x*+1, −*y*+2, −*z*+2; (iii) −*x*, −*y*+2, −*z*+2.
